# Supplementary material for: DeepQA: improving the estimation of single protein model quality with deep belief networks
Source: BMC Bioinformatics. 2016 Dec 5;17:495. doi: 10.1186/s12859-016-1405-y (PMC5139030; doi:10.1186/s12859-016-1405-y)
Supplement: Additional file 1: — Table S1. Z-score improvement of applying DeepQA for CASP11 top performance protein tertiary structure prediction methods. Table S2. TM-score and RMSD score (and their Z-score) of DeepQA on ab initio datasets. Table S3. TM-score and RMSD score of ProQ2 on ab initio datasets. Table S4. Average per-target correlation and loss for DeepQA and ResQ on 54 targets of CASP11. (DOCX 34 kb) [file 12859_2016_1405_MOESM1_ESM.docx]

**Supplementary Information**

*for*

DeepQA: Improving the estimation of single protein model quality with deep belief networks

Renzhi Cao^1^, Debswapna Bhattacharya^2^, Jie Hou^3^, and Jianlin Cheng^3, 4*^

^1^Department of Computer Science, Pacific Lutheran University, Tacoma, WA 98447, USA

^2^Department of Electrical Engineering and Computer Science, Wichita State University, Wichita, KS 67260, USA

^3^Department of Computer Science, University of Missouri, Columbia, MO 65211, USA

^4^Informatics Institute, University of Missouri, Columbia, MO 65211, USA

^*^To whom correspondence should be addressed. Phone: (573)-882-7306. Fax: (573)-882-8318. E-mail: chengji@missouri.edu.

| **Supplementary Item** | **Title** |
| --- | --- |
| Supplementary Table 1 | Z-score improvement of applying DeepQA for CASP11 top performance protein tertiary structure prediction methods |
| Supplementary Table 2 | TM-score and RMSD score of DeepQA on *ab initio* datasets. |
| Supplementary Table 3 | TM-score and RMSD score of ProQ2 on *ab initio* datasets. |

**Supplementary Table S1.** Z-score improvement of applying DeepQA for CASP11 top performance protein tertiary structure prediction methods

| Server name | Improvement on Z-score of GDT-TS score |
| --- | --- |
| Zhang-Server | 6.38 |
| QUARK | 5.93 |
| nns | -7.37 |
| myprotein-me | -1.46 |
| BAKER-ROSETTASERVER | 16.34 |
| MULTICOM-CONSTRUCT | -9.30 |
| MULTICOM-CLUSTER | 0.79 |
| TASSER-VMT | 11.46 |
| RaptorX | 6.66 |
| MULTICOM-NOVEL | 14.16 |

**Supplementary Table S2.** TM-score and RMSD score (and their Z-score) of DeepQA on ab initio datasets.

| Target name  (DeepQA) | TM-score  for top 1 | Z-score of  TM-score  for top 1 | RMSD for  top 1 | Z-score of  RMSD for  top 1 | TM-score  for best of  top 5 | Z-score of  TM-score  for top 5 | RMSD for  best of  top 5 | Z-score of  RMSD for  top 5 |
| --- | --- | --- | --- | --- | --- | --- | --- | --- |
| T0761 | 0.19 | -0.55 | 20.40 | 0.43 | 0.21 | 0.77 | 17.46 | -1.35 |
| T0763 | 0.18 | -0.52 | 20.50 | 1.80 | 0.23 | 1.70 | 14.78 | -2.05 |
| T0767 | 0.21 | 0.08 | 23.07 | 1.43 | 0.23 | 0.91 | 19.33 | -0.69 |
| T0771 | 0.21 | 0.81 | 19.19 | 0.05 | 0.22 | 1.46 | 16.87 | -1.41 |
| T0777 | 0.29 | 2.17 | 16.03 | -2.42 | 0.34 | 4.00 | 14.72 | -3.17 |
| T0781 | 0.22 | 1.73 | 21.59 | -1.36 | 0.22 | 1.73 | 21.59 | -1.36 |
| T0785 | 0.22 | 0.58 | 13.38 | -0.97 | 0.25 | 2.15 | 13.35 | -1.00 |
| T0789 | 0.25 | 0.87 | 19.98 | 0.48 | 0.27 | 1.65 | 18.12 | -0.57 |
| T0790 | 0.25 | 0.68 | 20.36 | 0.79 | 0.30 | 2.43 | 18.27 | -0.41 |
| T0791 | 0.27 | 2.63 | 16.53 | -2.15 | 0.30 | 3.95 | 15.08 | -3.00 |
| T0794 | 0.27 | 1.93 | 20.23 | -2.06 | 0.27 | 1.93 | 20.23 | -2.06 |
| T0806 | 0.25 | 0.48 | 16.89 | -0.65 | 0.26 | 0.64 | 15.06 | -1.78 |
| T0808 | 0.17 | -1.63 | 23.58 | 0.17 | 0.22 | 0.59 | 21.99 | -0.81 |
| T0810 | 0.25 | 1.01 | 24.85 | 1.01 | 0.25 | 1.14 | 22.14 | -0.37 |
| T0814 | 0.20 | 1.20 | 25.87 | 0.12 | 0.20 | 1.42 | 22.86 | -1.79 |
| T0820 | 0.23 | 1.06 | 14.01 | -2.19 | 0.26 | 1.97 | 14.01 | -2.19 |
| T0824 | 0.24 | 0.95 | 12.12 | -1.13 | 0.28 | 2.23 | 12.12 | -1.13 |
| T0827 | 0.23 | -0.15 | 21.34 | -0.19 | 0.29 | 1.41 | 17.64 | -1.48 |
| T0831 | 0.19 | 0.61 | 28.72 | -0.03 | 0.22 | 1.90 | 26.29 | -1.17 |
| T0832 | 0.24 | 0.46 | 19.49 | -0.14 | 0.25 | 1.08 | 17.56 | -1.33 |
| T0834 | 0.20 | 0.28 | 17.30 | -2.41 | 0.23 | 1.50 | 17.30 | -2.41 |
| T0836 | 0.21 | -0.75 | 19.38 | 0.68 | 0.29 | 1.89 | 17.01 | -0.47 |
| T0837 | 0.39 | 2.64 | 6.71 | -2.65 | 0.39 | 2.64 | 6.71 | -2.65 |
| T0855 | 0.27 | 0.49 | 14.69 | 0.48 | 0.31 | 1.72 | 10.82 | -1.82 |

**Supplementary Table S3.** TM-score and RMSD score of ProQ2 on ab initio datasets.

| Target name (ProQ2) | TM-score for top one | RMSD for top one | TM-score for best of top 5 | RMSD for best of top 5 |
| --- | --- | --- | --- | --- |
| T0834 | 0.18 | 23.64 | 0.23 | 21.32 |
| T0790 | 0.20 | 22.10 | 0.26 | 16.19 |
| T0824 | 0.21 | 14.29 | 0.25 | 11.99 |
| T0806 | 0.21 | 20.26 | 0.27 | 16.37 |
| T0767 | 0.21 | 23.07 | 0.24 | 17.92 |
| T0836 | 0.21 | 17.46 | 0.27 | 16.73 |
| T0791 | 0.24 | 20.18 | 0.25 | 17.19 |
| T0785 | 0.22 | 12.88 | 0.23 | 12.88 |
| T0855 | 0.29 | 11.04 | 0.30 | 10.82 |
| T0814 | 0.20 | 25.87 | 0.20 | 25.73 |
| T0781 | 0.18 | 24.17 | 0.21 | 23.26 |
| T0831 | 0.22 | 26.79 | 0.22 | 26.29 |
| T0789 | 0.23 | 18.04 | 0.25 | 18.04 |
| T0820 | 0.21 | 16.36 | 0.26 | 16.36 |
| T0808 | 0.21 | 23.80 | 0.22 | 21.99 |
| T0810 | 0.23 | 22.44 | 0.23 | 20.86 |
| T0771 | 0.17 | 21.18 | 0.20 | 18.15 |
| T0827 | 0.25 | 17.90 | 0.28 | 17.90 |
| T0761 | 0.21 | 20.22 | 0.21 | 18.96 |
| T0777 | 0.20 | 22.52 | 0.34 | 14.72 |
| T0832 | 0.24 | 18.39 | 0.25 | 17.81 |
| T0763 | 0.19 | 18.03 | 0.21 | 16.71 |
| T0794 | 0.27 | 20.23 | 0.27 | 20.23 |
| T0837 | 0.23 | 12.61 | 0.33 | 11.79 |

**Supplementary Table S4.** Average per-target correlation and loss for DeepQA and ResQ on 54 targets of CASP11.

|  | **ResQ** | | **ResQ** | | **DeepQA** | | **DeepQA** | |
| --- | --- | --- | --- | --- | --- | --- | --- | --- |
| **Target** | **Corr. on**  **stage 1** | **Loss on**  **stage 1** | **Corr. on**  **stage 2** | **Loss on**  **stage 2** | **Corr. on**  **stage 1** | **Loss on stage 1** | **Corr. on**  **stage 2** | **Loss on**  **stage 2** |
| T0760 | 0.93 | 0.01 | 0.72 | 0.02 | 0.61 | 0.34 | 0.40 | 0.02 |
| T0762 | 1.00 | 0.00 | 0.76 | 0.04 | 0.81 | 0.00 | 0.44 | 0.04 |
| T0763 | 0.06 | 0.06 | 0.16 | 0.06 | 0.26 | 0.03 | 0.14 | 0.02 |
| T0764 | 0.99 | 0.00 | 0.93 | 0.01 | 0.88 | 0.00 | 0.44 | 0.00 |
| T0765 | 0.39 | 0.00 | -0.47 | 0.48 | 0.82 | 0.00 | 0.25 | 0.29 |
| T0766 | 0.99 | 0.00 | 0.99 | 0.01 | 0.47 | 0.49 | 0.67 | 0.01 |
| T0768 | 0.92 | 0.21 | 0.95 | 0.10 | 0.67 | 0.36 | 0.52 | 0.00 |
| T0769 | 0.91 | 0.00 | 0.33 | 0.21 | 0.78 | 0.00 | 0.57 | 0.09 |
| T0770 | 0.94 | 0.02 | 0.61 | 0.05 | 0.88 | 0.02 | 0.53 | 0.03 |
| T0771 | -0.57 | 0.08 | -0.06 | 0.12 | 0.29 | 0.08 | 0.35 | 0.00 |
| T0772 | 0.98 | 0.00 | 0.57 | 0.03 | 0.79 | 0.09 | 0.57 | 0.02 |
| T0773 | 0.67 | 0.23 | 0.77 | 0.18 | 0.69 | 0.00 | 0.63 | 0.24 |
| T0774 | 0.86 | 0.00 | 0.83 | 0.06 | 0.29 | 0.15 | 0.42 | 0.02 |
| T0776 | 0.98 | 0.00 | 0.75 | 0.03 | 0.77 | 0.13 | 0.41 | 0.03 |
| T0777 | -0.41 | 0.07 | 0.39 | 0.06 | 0.70 | 0.03 | 0.62 | 0.04 |
| T0782 | 0.84 | 0.00 | 0.86 | 0.20 | 0.32 | 0.22 | 0.30 | 0.20 |
| T0784 | 0.98 | 0.00 | 0.90 | 0.07 | 0.53 | 0.43 | 0.14 | 0.02 |
| T0785 | -0.12 | 0.11 | -0.05 | 0.08 | 0.67 | 0.05 | 0.48 | 0.09 |
| T0786 | 0.95 | 0.00 | 0.84 | 0.07 | 0.44 | 0.33 | 0.71 | 0.03 |
| T0792 | 0.96 | 0.00 | 0.43 | 0.12 | 0.50 | 0.29 | 0.57 | 0.02 |
| T0796 | 0.84 | 0.00 | 0.89 | 0.07 | 0.75 | 0.26 | 0.54 | 0.03 |
| T0800 | 0.68 | 0.00 | 0.74 | 0.11 | 0.70 | 0.25 | 0.38 | 0.01 |
| T0801 | 0.99 | 0.00 | 0.64 | 0.03 | 0.90 | 0.00 | 0.67 | 0.02 |
| T0803 | 0.95 | 0.00 | 0.88 | 0.08 | 0.31 | 0.13 | 0.07 | 0.08 |
| T0805 | 0.99 | 0.00 | 0.79 | 0.02 | 0.64 | 0.23 | 0.44 | 0.09 |
| T0806 | -0.29 | 0.07 | -0.11 | 0.14 | 0.79 | 0.00 | 0.45 | 0.01 |
| T0807 | 0.99 | 0.08 | 0.68 | 0.06 | 0.95 | 0.11 | 0.70 | 0.03 |
| T0811 | 0.99 | 0.00 | 0.77 | 0.01 | 0.77 | 0.00 | 0.58 | 0.01 |
| T0812 | 0.65 | 0.00 | 0.96 | 0.03 | 0.32 | 0.23 | 0.45 | 0.00 |
| T0813 | 0.96 | 0.00 | 0.40 | 0.05 | 0.58 | 0.27 | 0.23 | 0.02 |
| T0815 | 0.99 | 0.00 | 0.81 | 0.03 | 0.84 | 0.01 | 0.44 | 0.08 |
| T0816 | -0.68 | 0.45 | -0.42 | 0.35 | 0.79 | 0.00 | 0.49 | 0.21 |
| T0818 | 0.70 | 0.00 | 0.69 | 0.03 | 0.42 | 0.00 | 0.09 | 0.02 |
| T0819 | 0.99 | 0.00 | 0.85 | 0.05 | 0.87 | 0.16 | 0.64 | 0.04 |
| T0821 | 0.36 | 0.22 | 0.88 | 0.07 | 0.64 | 0.00 | 0.67 | 0.03 |
| T0822 | -0.06 | 0.25 | 0.91 | 0.07 | 0.30 | 0.21 | 0.07 | 0.34 |
| T0823 | 0.99 | 0.00 | 0.60 | 0.03 | 0.89 | 0.05 | 0.66 | 0.00 |
| T0824 | 0.20 | 0.04 | 0.47 | 0.02 | 0.38 | 0.11 | 0.14 | 0.02 |
| T0829 | 0.85 | 0.00 | 0.51 | 0.24 | 0.85 | 0.00 | 0.64 | 0.00 |
| T0832 | -0.28 | 0.02 | -0.08 | 0.11 | 0.64 | 0.01 | 0.44 | 0.07 |
| T0833 | 0.98 | 0.00 | 0.89 | 0.04 | 0.49 | 0.00 | 0.44 | 0.02 |
| T0835 | 0.96 | 0.00 | 0.80 | 0.07 | 0.74 | 0.09 | 0.72 | 0.01 |
| T0836 | -0.35 | 0.09 | 0.16 | 0.09 | 0.45 | 0.03 | 0.46 | 0.07 |
| T0837 | 0.49 | 0.00 | -0.21 | 0.27 | 0.81 | 0.02 | 0.65 | 0.04 |
| T0838 | 0.72 | 0.00 | 0.90 | 0.00 | 0.83 | 0.00 | 0.54 | 0.00 |
| T0841 | 0.99 | 0.00 | 0.89 | 0.00 | 0.81 | 0.00 | 0.76 | 0.01 |
| T0843 | 0.98 | 0.06 | 0.67 | 0.02 | 0.94 | 0.16 | 0.44 | 0.03 |
| T0847 | 0.97 | 0.00 | 0.46 | 0.03 | 0.83 | 0.00 | 0.23 | 0.06 |
| T0849 | 0.98 | 0.08 | 0.74 | 0.01 | 0.46 | 0.28 | 0.42 | 0.08 |
| T0851 | 0.96 | 0.16 | 0.85 | 0.04 | 0.59 | 0.18 | 0.52 | 0.05 |
| T0855 | 0.38 | 0.19 | -0.40 | 0.26 | 0.74 | 0.00 | 0.64 | 0.13 |
| T0856 | 1.00 | 0.00 | 0.69 | 0.07 | 0.87 | 0.06 | 0.34 | 0.06 |
| T0857 | 0.88 | 0.00 | 0.94 | 0.07 | 0.42 | 0.25 | 0.04 | 0.30 |
| T0858 | 0.99 | 0.00 | 0.74 | 0.04 | 0.88 | 0.00 | 0.62 | 0.03 |
| Mean | 0.67 | 0.05 | 0.58 | 0.09 | 0.65 | 0.11 | 0.46 | 0.06 |
